# Posttranslational modifications: an emerging functional layer of diet-host-microbe interactions

**DOI:** 10.1128/mbio.02387-24

**Published:** 2024-09-10

**Authors:** Lirit Duchovni, Genrieta Shmunis, Lior Lobel

**Affiliations:** 1 The Goodman Faculty of Life Sciences, Bar-Ilan University, Ramat Gan, Israel; The Ohio State University, Columbus, Ohio, USA

**Keywords:** diet-microbe-host interactions, PTM, metaproteomics, proteomics, host-microbe interactions

## Abstract

The microbiome plays a vital role in human health, with changes in its composition impacting various aspects of the body. Posttranslational modification (PTM) regulates protein activity by attaching chemical groups to amino acids in an enzymatic or non-enzymatic manner. PTMs offer fast and dynamic regulation of protein expression and can be influenced by specific dietary components that induce PTM events in gut microbiomes and their hosts. PTMs on microbiome proteins have been found to contribute to host-microbe interactions. For example, in *Escherichia coli*, S-sulfhydration of tryptophanase regulates uremic toxin production and chronic kidney disease in mice. On a broader microbial scale, the microbiomes of patients with inflammatory bowel disease exhibit distinct PTM patterns in their metaproteomes. Moreover, pathogens and commensals can alter host PTM profiles through protein secretion and diet-regulated metabolic shifts. The emerging field of metaPTMomics focuses on understanding PTM profiles in the microbiota, their association with lifestyle factors like diet, and their functional effects on host-microbe interactions.

## INTRODUCTION

According to the central dogma of biology, the genetic material, DNA, is amplified and transcribed into RNA molecules that undergo modifications affecting their sequence, stability, and function (1, 2). Polysomes translate coding sequences on RNA molecules into proteins, biology’s main biochemical and structural effectors. This genetic information flow results in amplification and diversification of the effector molecules. For example, the human genome has ~20,000 DNA-coding sequences, and splice variants raise the number of unique coding RNAs to ~70,000 protein isoforms (3). Moreover, on top of the genetic regulation lies a layer of biochemical regulation termed posttranslation modification (PTM) (3). There are hundreds of known PTMs that occur on amino acid side chains, varying from the addition of small chemical groups such as phosphate and acetate, to the incorporation of small proteins, e.g., ubiquitin in eukaryotes and prokaryotic ubiquitin-like protein (Pup) in bacteria (4). PTM regulation of human proteins increases the potential space of human proteoforms to at least the range of ~10^6^ (3). Thus, understanding the profile, function, and dynamics of PTMs is crucial for deciphering the biochemical state of cells.

The cellular environment, modulated by the extracellular one, is the central modulator of PTMs, most of which occur in the time scale of seconds to minutes (5). Thus, PTM regulation is highly complex and significantly faster than gene regulation at the DNA or RNA levels. Certain PTMs are added or removed by specialized enzymes. For example, phosphorylation, perhaps the best-studied PTM, is mediated by various protein-specific kinases that attach a phosphate group to the hydroxyl of serine, threonine, or tyrosine side chains, while specific phosphatases remove it (6). Some PTMs can occur spontaneously by chemical reactions with reactive species, such as oxidation and S-sulfhydration or S-nitrosylation of cysteine residues by reactive oxygen or reactive nitrogen species, respectively (7, 8). Conversely, thioreductases remove the oxygen, sulfide, or nitrate groups. Other PTMs can occur via both enzymatic and spontaneous reactions, e.g., lysine acetylation, where acetyltransferases add acyl groups and deacetylases remove them, while acetyl-CoA or acetyl-phosphate can directly acetylate lysines (9).

Most PTMs have been well studied in eukaryotic systems, revealing their immense scale and diverse functions in cell signaling and metabolism (4). In contrast, the role of PTMs in bacterial physiology is much less studied (10). However, PTMs are fundamental in bacteria and have been the subject of increased interest. For an elegant and comprehensive review of PTMs in human-associated bacteria, see Bastos et al. (11). This minireview, instead, focuses on the emerging field of diet-microbe-PTM interactions, which may play a role in dietary effects on host phenotypes, especially when there are no overt alterations to the microbiome’s composition.

## DIETARY-RELATED PTMS

The gut microbiota is the consortium of bacteria, archaea, fungi, protists, and viruses that inhabit the host’s gastrointestinal tract (12). Among several lifestyle factors, diet is the principal regulator of the microbiota’s composition and activity (13, 14). Considering that diet modulates host PTM patterns (15, 16) and that many dietary components reach the colon-resident bacteria, it is apparent that dietary-induced PTMs can modulate bacterial activity. The molecular mechanisms by which diet asserts its effects are presumed to be multifaceted. One established mechanism involves favoring the metabolic capabilities and, hence, the growth of certain species, resulting in their outgrowth at the expense of other species that are less adjusted to the host’s dietary regimen (17). Since this mechanism includes changes in anaerobic bacteria growth, the time scale of the effect can be days to weeks. Recently, we provided proof-of-concept for a more rapid mechanism of dietary-induced microbial activity modulation that does not include a shift in microbial composition but rather a direct effect on its activity. The effect is mediated via the posttranslational modification S-sulfhydration (see below) on a bacterial enzyme cysteine residue (18). To explore more such scenarios, we address several PTMs that may be modulated by diet.

### Cysteine S-Sulfhydration

Hydrogen sulfide (H_2_S) is a highly reactive gasotransmitter in animal physiology, akin to nitric oxide and carbon monoxide (19). One way H_2_S signals is through protein S-sulfhydration, which occurs at reactive cysteine residues. S-Sulfhydration occurs via a nucleophilic attack of a reactive sulfur species, generated by an H_2_S reaction with polysulfides on the side chain of a cysteine residue, resulting in the R-SH converting to R-S-SH (20). The cysteine residue’s acid dissociation constant (pKa) determines its reactivity and susceptibility to S-sulfhydration. Sulfhydrated proteins/peptides can be detected using a biotin-labeled maleimide pull-down assay (21), which interacts specifically with sulfhydryl groups of native or S-sulfhydrated cysteine residues but does not affect nitrosylated or oxidized cysteine residues. The release of S-sulfhydrated proteins/peptides from the streptavidin beads is specific to S-sulfhydrated cysteines due to the disulfide bond reduction that is found in the R-S-S-maleimide bond, but not in the R-S-maleimide bond (21).

S-sulfhydration was shown to regulate many cellular functions in eukaryotic cells. For example, the primary transcription factor NF-κB was shown to be activated by S-sulfhydration (22), while GAPDH S-sulfhydration inactivates the protein’s function (23). However, little is known about protein S-sulfhydration in bacteria, the S-sulfhydrome analyses of *Escherichia coli,* and *Staphylococcus aureus* being the exception (18, 24). Nonetheless, anecdotal studies found bacterial transcription factors regulated by S-sulfhydration (25–30).

The level of H_2_S in the colon is controlled by the activity of the resident microbiota (18, 31), as germ-free animals have very little H_2_S in their gut and stool. In humans, it was shown that dietary protein consumption directly correlates with stool H_2_S production (32). Thus, diet affects the levels of protein S-sulfhydration in the colon (18).

### Lysine (K) acetylation

The best-studied gut bacterial metabolic products are the dietary fiber-derived short-chain fatty acids (SCFAs) that have multiple pleiotropic effects on the host’s physiology, regulating immune cell function and enterocyte metabolism (33, 34). Bacterial fermentation of dietary fibers, such as pectin and inulin, occurs solely by bacteria in the gastrointestinal tract, producing high amounts of SCFA (34). The most abundant SCFA in the colon is acetate, and its levels can influence the abundance of the acetyl donors acetyl-coenzyme A and acetyl phosphate (AcP) that are crucial for acetylating lysine residues in bacteria (35, 36). K-acetylation is an acetyl group’s enzymatic or spontaneous addition to a lysine epsilon(ε)-amino group (37) and, as such, is a widespread PTM in all organisms. K-acetylation is detected using affinity purification with antibodies specific to acetylated lysines.

K-acetylation neutralizes the positive charge of the lysine sidechain’s amino group, resulting in a neutral charge that inhibits its reactivity and affects protein function. K-acetylation affects many cellular functions; in eukaryotes, K-acetylation of histone proteins decreases their affinity to DNA and increases transcription factor binding (38). In bacteria, K-acetylation also affects various bacterial proteins. For example, K-acetylation of several central metabolism bacterial enzymes, specifically of fatty acid metabolism and the tricarboxylic acid (TCA) cycle, is evolutionarily conserved and was shown to regulate their activity as a switch-off signal (39). K-acetylation can also regulate more specialized functions, such as quorum signaling in *Pseudomonas aeruginosa* (40).

**Fig 1:**
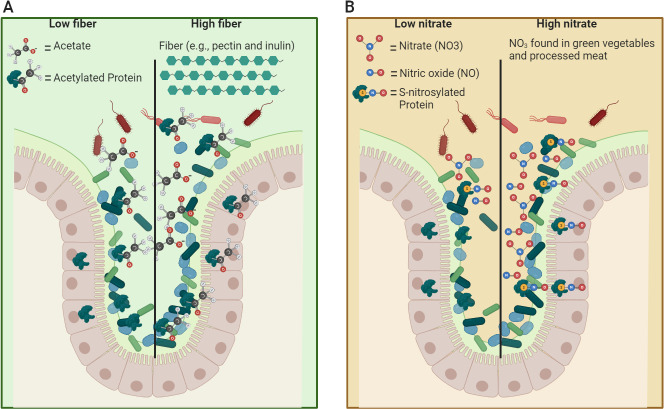
PTMs are proposed to be induced by diet. (A) Soluble dietary fiber fermentation in the colon increases acetate levels, which may augment protein K-acetylation in bacteria and host cells and affect their metabolism and signaling. (B) Dietary nitrate can be reduced to nitric oxide (NO) in the colon by bacterial respiration in anaerobic conditions. NO contributes to RNS generation by S-nitrosylating proteins on their native cysteine residues, regulating their function. These illustrations were created with BioRender.com.

Modulating dietary fiber intake may lead to differential colonic acetate and AcP levels, resulting in increased non-enzymatic protein acetylation in the microbiome and host cells on a high-fiber diet (Fig. 1A). Therefore, we speculate that some associations found between dietary fiber and host phenotypes are driven by K-acetylation modulation, particularly in cases where no clear changes in the gut microbiome composition were observed. In support of this hypothesis, germ-free mice had fewer acetylated proteins in their colonic epithelial cells than conventionally reared mice with complex microbiota (41). Additionally, gnotobiotic mice were found to have distinct acetylation and succinylation in their hippocampus compared with mice with conventional microbiota (42, 43). Furthermore, the acetylation profile of histones in host tissues, detected using a mass-spectrometry method for lysine derivatization, depends on dietary fiber-modulated gut bacterial SCFA production (15). Hence, K-acetylation in both the microbiome and host cells can be regulated by dietary fiber.

### S-nitrosylation

Cysteine S-nitrosylation is one of the many PTMs that can decorate a cysteine’s thiol group. S-nitrosylation is a PTM that occurs non-enzymatically when a reactive nitrogen species (RNS) reacts with a cysteine sidechain’s thiol (−SH), resulting in the formation of a nitrosothiol (R-SNO) (7). S-nitrosylated proteins/peptides are detected using a biotin switch assay (44), where native or persulfated cysteine residues are blocked using an alkylating agent such as iodoacetamide. Then, ascorbate is used to specifically reduce S-nitrosylated residues and allow them to be labeled with a biotin-maleimide tag, followed by affinity purification using streptavidin beads.

A significant contributor to RNS formation is the gasotransmitter NO (20). NO has various known effects on human physiology, from regulation of vasoconstriction to brain signaling; some NO effects are mediated by S-nitrosylation (7). As with S-sulfhydration, the effects of S-nitrosylation are pleiotropic, meaning they can inhibit or enhance a protein’s function. Notable examples of S-nitrosylation include the NMDA-type glutamate receptor, whose R-SNO form exerts neuroprotective effects on mammalian brain cells (45–47). In bacteria, S-nitrosylation was shown to regulate the OxyR transcription factor, serving as a redox switch for activating its nitrosative stress-protective activity during nitrate respiration in anaerobic growth, while oxidation of the same cysteine residue causes OxyR to regulate a different set of genes (48). In *E. coli*, an enzymatic complex was reported to regulate transnitrosylation. The hybrid cluster protein (Hcp) becomes S-nitrosylated on specific cysteine residues following NO production by NarGHI, the NO synthase complex. Hcp, in turn, functions as a nitrosothiol donor to the various proteins it can directly interact with, and these may further facilitate transnitrosylation (49). This study demonstrated that protein interactions can regulate S-nitrosylation in bacteria. As with other PTMs, S-nitrosylation was found to regulate essential metabolic enzymes in *Mycobacterium tuberculosis*, inhibiting dihydrolipoamide dehydrogenase, crucial for its lipid metabolism (50).

In the dietary context, nitrate and nitrite, abundant in green leafy vegetables and processed meat, can be reduced to NO by various oral and gut bacteria (51, 52). Thus, we and others propose that dietary nitrate levels can modulate gut NO concentrations and alter protein S-nitrosylation in the host and its microbiome (Fig. 1B). Interestingly, it was reported that *Caenorhabditis elegans*-fed NO-producing bacteria had increased longevity compared with worms fed non-NO-producing mutants (53). Furthermore, increased S-nitrosylation was observed in *C. elegans*-fed NO-producing bacteria (54, 55), implicating a potential molecular mechanism for the former finding. Integrating the findings of these studies supports our premise that diet and the microbiota can affect host physiology via modulation of S-nitrosylation.

## CHALLENGES IN metaPTMomics

The study of PTMs in complex microbial communities, i.e., environmental and host-associated microbiomes, is still in its early stages. The first metaPTMomics analysis was published in 2014 and described eight PTM profiles, including S-nitrosylation and K-acetylation, in two growth stages of mine drainage site microbiomes (56). The authors found CRISPR-associated proteins (Cas) to have multiple PTMs, an aspect of Cas activity still understudied. A second environmental microbiome metaPTMomics study was published in 2016, looking into seven PTMs, including K-acetylation and S-nitrosylation, and identified more than a thousand unique PTM sites in multiple bacterial taxa in a hydrothermal vent microbial community (57). In both environmental microbiome studies, multiple central metabolism enzymes were modified, which implies that PTMs can rapidly regulate metabolic fluxes at the community level.

Studies in the more complex human microbiome community identified acetylation and succinylation patterns associated with dysbioses in patients with inflammatory bowel disease (IBD) (58, 59). In a first-ever attempt to map a PTM profile of the gut microbiome, Zhang et al. identified 136 K-acetylation sites from diverse taxa, mainly on metabolic enzymes and translational machinery proteins, that vary significantly in the stool microbiomes of IBD patients versus healthy controls (58). In a follow-up study, they expanded their research to K-succinylation and K-propionylation profiles (59). These two modifications turn the positive charge of the amino group into a negatively charged one (60).

In the following sections, we summarize current challenges and limitations in metaPTMomics and suggest some solutions.

### Multiple proteomes

Most of the research on bacterial PTMs has been done in monocultures due to the ease of culturing and proteomics analysis of PTMs in a single organism. To propel metaproteomic research into exploring metaPTMomics, several challenges must be overcome. The most common process for identifying mass spectra is matching the actual spectra to *in silico*-simulated spectra with sophisticated search engines (61). This type of analysis is computationally and statistically complicated in metaproteomic searches since there is a vastly inflated peptide search space, where the database contains millions of proteins instead of tens of thousands in single organism searches. This is further aggravated when variable PTMs are added to the search. Due to the expanded peptide database, the chances of randomly finding a peptide-spectrum match (PSM) that is incorrectly assigned increase dramatically, as does the overall computational runtime (62). The inflated database results, in part, from homology between bacterial proteins. For example, clustering the unified human gut proteome at 100% identity results in around 173 million proteins, while a 90% cutoff results in ~14 million protein clusters (63). Enforcing a lower cutoff will reduce the unique protein clusters and ease the analyses; however, it will significantly hinder taxonomic resolution and could result in grouping functionally distinct proteins. One solution to the database size issue is to use a two-step searching strategy; in the first step, the metaproteome is surveyed to identify which bacteria are present, and then, a specific database containing their proteomes is constructed (64). Another regularly used option is to match the metaproteomic data with shotgun metagenomic data (65, 66). In this scenario, the complete set of DNA-coding sequences can be determined from the DNA data and then used to compile a database for the PSM analysis. A cheaper but less accurate alternative is to use 16S rDNA sequencing to infer the bacterial taxa present and compile a metaproteome from available closely related genomes *in silico*. However, these methods are expensive and require more sample biomass for DNA analyses. Although improvements in the field have significantly increased the throughput of mass spectrometry, allowing the processing of ~200 metaproteomic samples per week (67), there is a great need for novel computational and statistical frameworks to ease and augment metaproteomic analysis.

### Enrichment

Most PTMs occur at low stoichiometry ratios, meaning that expensive and laborious methods are needed to enrich each PTM (68), which requires a large sample biomass, usually 1 mg of protein or more. Potential solutions include the use of enrichment methods in a serial manner (e.g., first enrich for phosphorylation and process the flowthrough for K-acetylation pull-down) (69) or developing higher coverage mass-spectrometry techniques to allow direct identification of PTMs without enrichment but keeping the standard runtime windows to allow high-throughput analysis. As a step in this direction, an environmental metaproteomic study identified eight PTM profiles without enrichment using a long 22-hour liquid chrormatography tandem mass spectometry (LC-MS/MS) run on an LTQ Orbitrap mass spectrometer (56). The authors probed eight distinct PTMs simultaneously, using a supercomputer with 35,000 central processing units, and obtained profiles for each PTM in two distinct growth phases of an acid mine drainage biofilm growth. However, this analysis required enormous computational resources.

### PTM crosstalk

The fact that hundreds of known PTMs occur on just half of the proteinogenic amino acids results in significant overlap and crosstalk between PTMs with distinct biological outcomes. For example, acetylation of lysine neutralizes the positive charge of the amine group, while succinylation results in a negative charge (60), having divergent biochemical consequences to the protein’s function. Highlighting this is a study on the effects of ultraviolet stress on acetylation and succinylation profiles in HeLa cells, which revealed that malate dehydrogenase (MDH2) is differentially acylated (i.e., acetylated vs. succinylated) following DNA damage, implying that each PTM has a different functional regulation (70). Similarly, S-nitrosylation and S-sulfhydration, both occurring on cysteine side chain thiols, compete and have different functional outcomes. For example, the S-sulfhydration of endothelial nitric oxide synthase enhances its activity by inducing its phosphorylation and dimerization, while S-nitrosylation decreases the activity by favoring the non-active monomeric form (71). In bacteria, the case of OxyR mentioned above is an example of a competition between oxidation to sulfonic acid and S-nitrosylation of the same cysteine residue that results in distinct gene expression (48).

Some PTMs may affect the co-occurrence of other PTMs, for example, a specific serine/threonine phosphorylation motif excludes acetylation on a nearby lysine (72). Therefore, it is highly recommended that combined enrichments be performed for PTMs of interest, such as ones induced by a common dietary component, to get an overall view of the phenotype.

### Dynamics

The dynamic nature of PTMs is much faster than changes at the DNA or RNA levels or even the protein translation level, due to the short half-life of reactive oxygen, nitrogen, and sulfur species and the rapid action of enzymes that catalyze the addition or removal of PTMs. Therefore, one should work prudently to preserve the natural state of the sample, avoiding adding or removing biological signals. If oxidation of PTMs is of concern, anaerobic conditions (e.g., working in a glove box and degassing of buffers) should be considered. Alternatively, molecules that fix the PTM status can mitigate signal loss. For example, maleimide addition to the lysis buffer will preserve S-sulfhydration events, even in oxygenic conditions. Moreover, we must be mindful about taking a snapshot of a metaproteome and, if possible, repeatedly measure the same environment, be it a hydrothermal vent or a person’s gut microbiome. An elegant study used time series analyses of protein synthesis and degradation in immune cells, revealing novel insights into the role of HSP90 chaperone in proteostasis (73). In microbes, the changes to the yeast proteome during the transition from exponential growth to stationary phase revealed gross differences in proteome changes between aerobic and anaerobic conditions, with far fewer changes in anaerobic growth, implying that anaerobic cells are less adaptable to starvation (74). The best-studied bacterial PTMome dynamics are that of *E. coli*, where several PTMs were studied during various growth phases and medium conditions to reveal novel PTMs and their role in protein degradation (75). In contrast to specific species’ PTMome dynamics, little is known about the dynamics of metaPTMomes. For example, a scenario comparing the metaPTMomes of individuals during a dietary-fiber intervention study should cover multiple time points. Of course, such a strategy comes with a monetary burden and a need for sophisticated statistical analyses, but it would be instrumental in elucidating novel PTM-related molecular mechanisms.

## PROPOSED ROLE OF PTMS IN DIET-HOST-MICROBIOTA INTERACTIONS

As mentioned above, in diet-microbiome studies, especially ones that investigate the role of SCFA in modulating the host phenotype, PTMs may play a role in these three-way interactions. For example, a human clinical trial found that a Microbiome Enhancer Diet that increased bacterial fermentation and SCFA production in the colon correlated with lower metabolized energy for the host and reduced weight (76). The rationale behind the phenomenon is assumed to be related to increased bacterial harvesting of the host’s diet; however, the molecular mechanisms that drive it may include acylation (e.g., acetylation and propionylation) of bacterial and host proteins. Similarly, the role of protein acylation in the metaproteome of mice deprived of dietary fiber can help elucidate the aggravation of food allergy by *Akkermansia muciniphila* (77), as currently it is hypothesized that fiber deprivation results in the expansion of mucus-degrading bacteria that disrupt the gut barrier and break immune tolerance, but K-acetylation could also play a role in either the bacterial or host proteome functions. Furthermore, enteric pathogens are known to benefit from reactive oxygen and nitrogen species production during gut inflammation or due to imbalanced diets (78, 79). Although the pathogens’ increased survival and fitness are mainly attributed to aerotolerance and reactive species harvesting, it is plausible that oxidative modifications (e.g., sulfonation and nitrosylation) to their proteomes could enhance their pathogenicity. Regarding S-nitrosylation, we are intrigued by the correlation between dietary nitrate intake and the abundance of the bacterial metabolites trimethylamine N-oxide (TMAO) and kynurenine (80). Microbially produced TMAO is known to contribute to atherosclerosis (81). Thus, it is tempting to speculate that bacteria nitrate reduction and S-nitrosylation may play a role in TMAO production and host cardiovascular disease. Moreover, microbe interactions with complex diets, such as the Mediterranean diet, Western-type diet, ketogenic diet, and high-protein diet, can benefit from metaPTMomic analyses.

**Fig 2:**
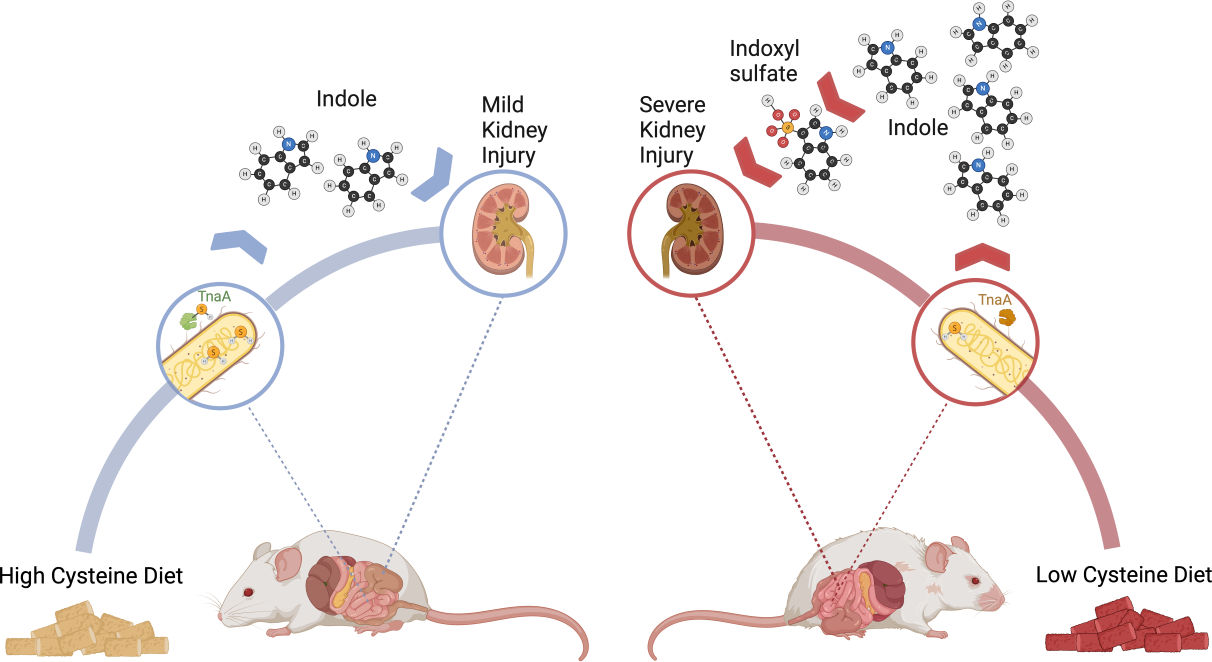
Illustration showing the effects of low- and high-cysteine diets on chronic kidney disease progression. A high-cysteine diet leads to increased gut H_2_S levels and microbial tryptophanase S-sulfhydration, which inhibits indole production and results in lower serum indoxyl-sulfate levels and milder progression of chronic kidney disease mice (adapted from reference 18). The image was created with BioRender.com.

Lastly, we wish to review the most detailed diet-host-microbe PTM interaction. Dietary cysteine is known to increase colonic H_2_S concentrations via bacterial cysteine degradation (32). Thus, we speculated that dietary cysteine may affect gut bacterial protein S-sulfhydration. As mentioned above, S-sulfhydration is a non-enzymatic PTM that is induced by H_2_S and plays a role in cellular signaling and metabolic regulation (82). Indeed, the bacterial enzyme tryptophanase (TnaA) was found to be S-sulfhydrated in the mouse gut in a dietary-related context. A high cysteine diet resulted in the inhibition of TnaA activity and decreased the breakdown of tryptophan into pyruvate, ammonium, and indole; the latter is oxidized in the liver, forming indoxyl sulfate, a uremic toxin that injures the kidney and reduces renal function (Fig. 2) (18). In a mouse model of chronic kidney disease, dietary cysteine modulated the disease’s progression, with mice fed a high cysteine diet having improved clinical outcomes (18). *In vivo* analyses revealed that TnaA is differentially S-sulfhydrated depending on dietary cysteine levels, and its modification is negatively correlated with blood indoxyl sulfate levels. No changes in the microbiota composition were observed, implying that the mechanism is mainly driven via dietary-induced S-sulfhydration.

## CONCLUSIONS

PTMs are emerging as important regulators of host-microbe interactions (83); however, most cases were inferred from single organism proteomes, while only a handful of metaPTMomics studies have been published (56–59). We envisage that with looming advances in metaproteomics and PTM analyses, we will see a rush of microbiome PTMomics studies that will bring us closer to understanding the mechanisms of (diet)-microbe-host interactions. We suggest that at least three dietary-induced PTMs play a role in this three-way interaction, but other PTMs, such as methylation and glycosylation, may be affected by diet. On the other hand, the gut microbiota’s modulation of host cells’ PTM profiles has garnered attention, and future experiments with gnotobiotic mice or dietary intervention studies will reveal the extent and dependence of host PTMs on bacterial activity. With the advancement of mass spectrometry and computational analysis tools, we trust that PTMs will emerge as key players in host-microbiome interactions that may be altered to ameliorate dysbiosis.
